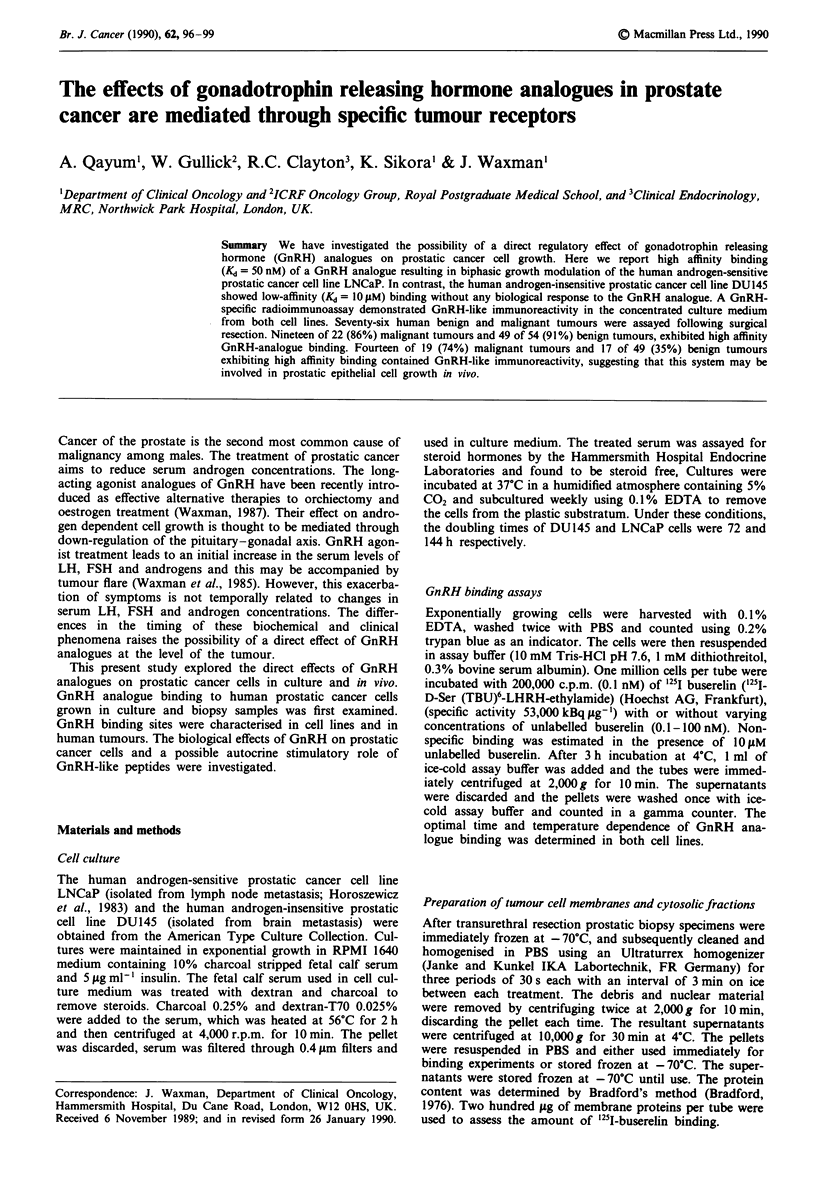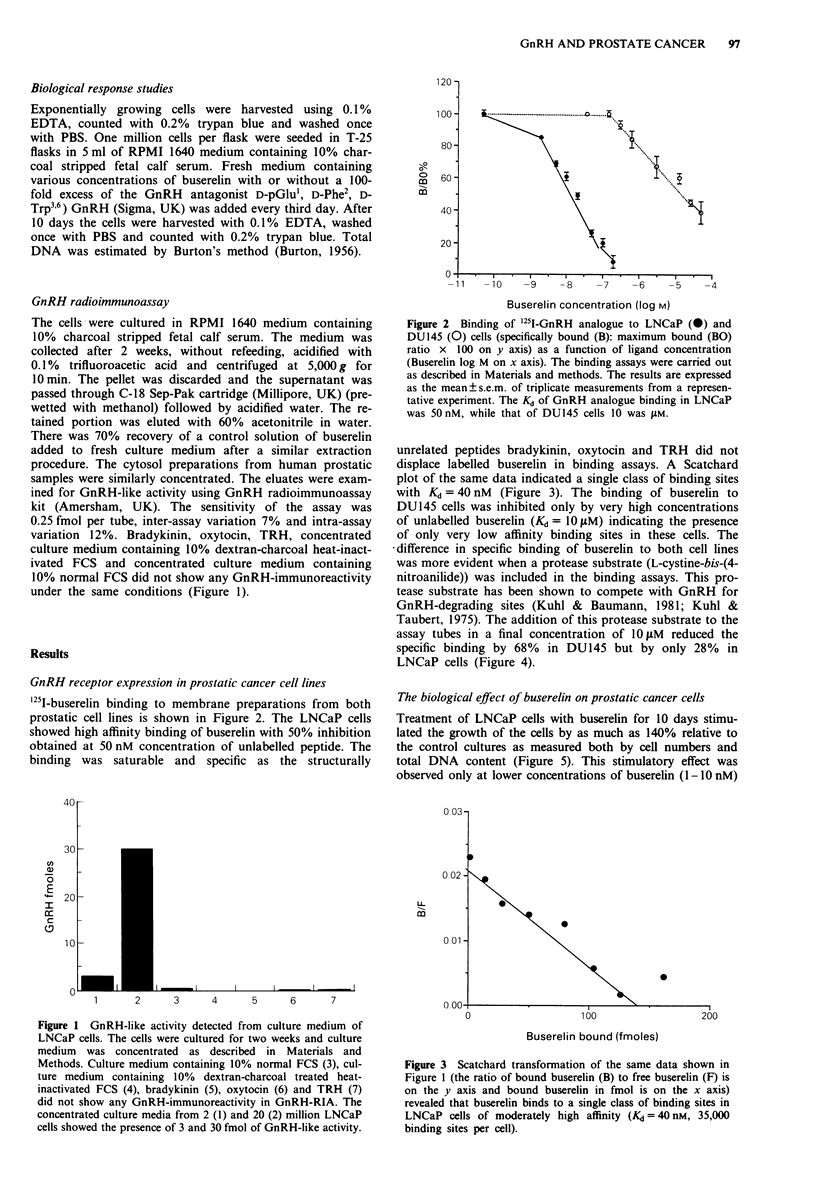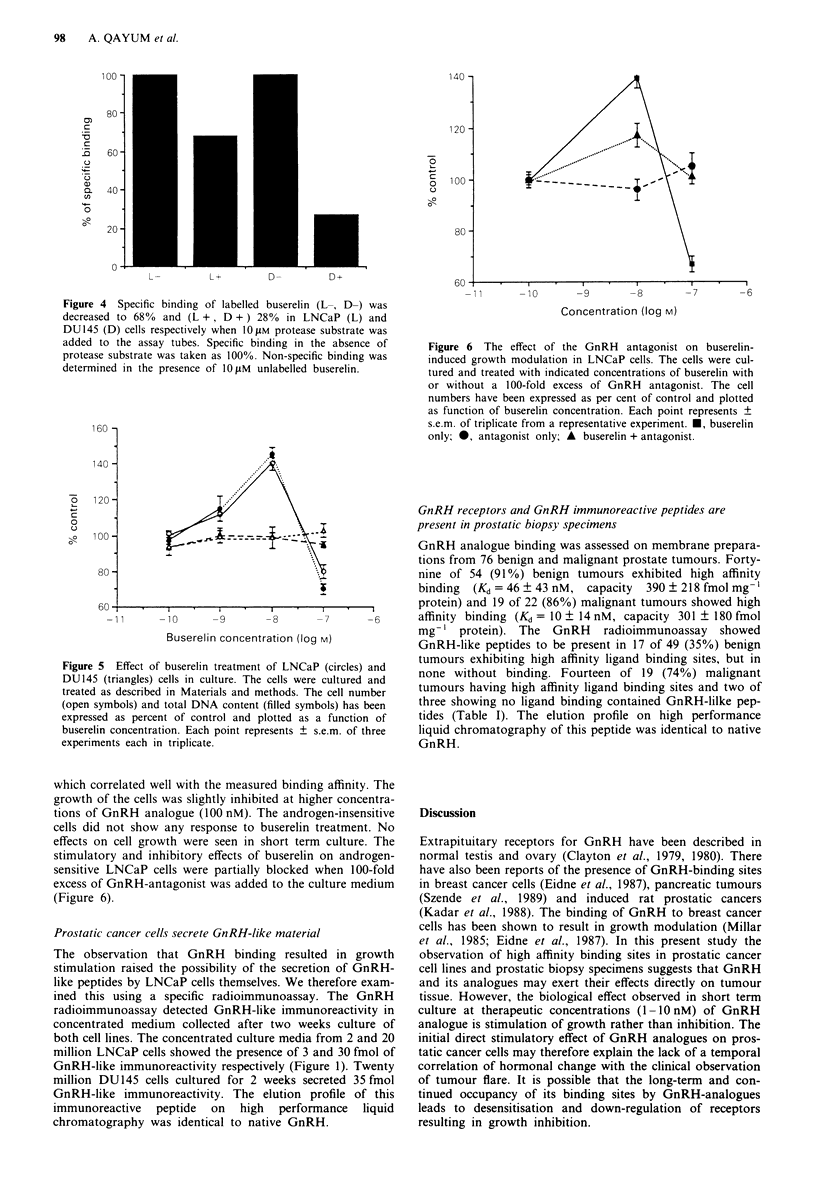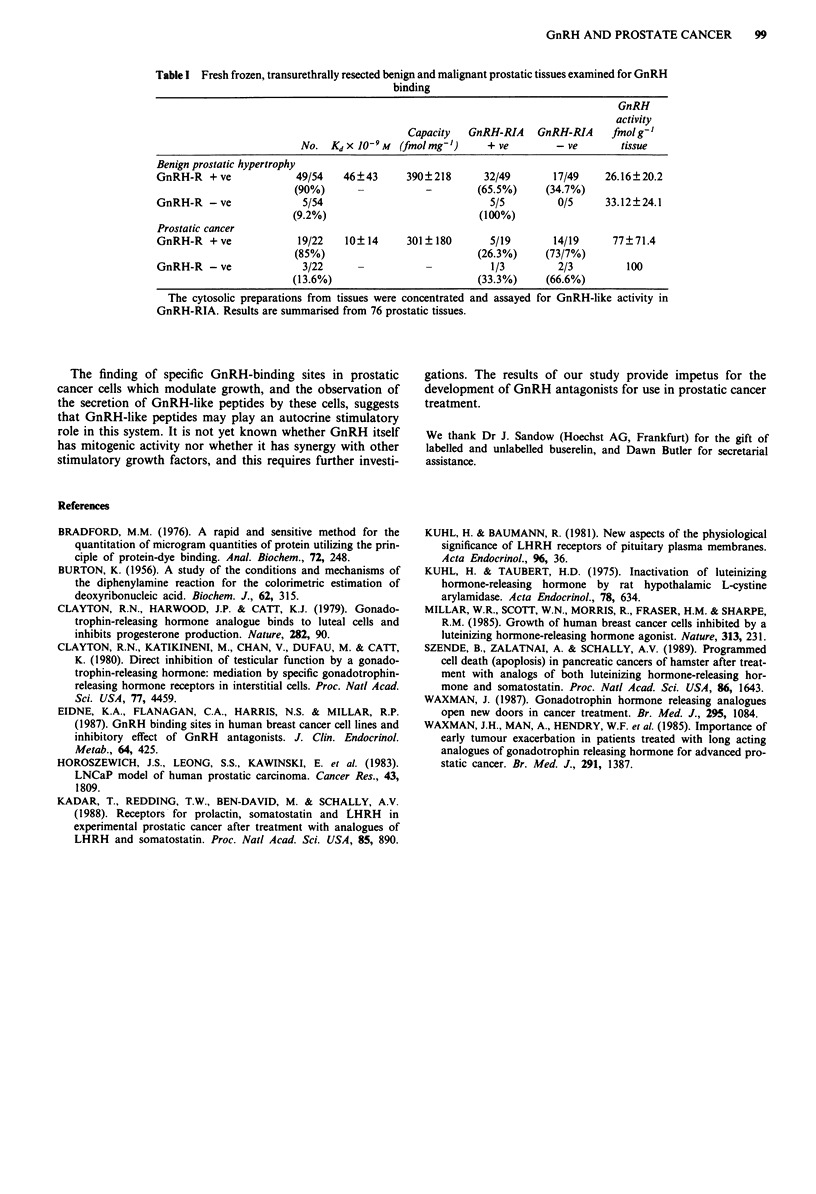# The effects of gonadotrophin releasing hormone analogues in prostate cancer are mediated through specific tumour receptors.

**DOI:** 10.1038/bjc.1990.236

**Published:** 1990-07

**Authors:** A. Qayum, W. Gullick, R. C. Clayton, K. Sikora, J. Waxman

**Affiliations:** Department of Clinical Oncology, Royal Postgraduate Medical School, London, UK.

## Abstract

We have investigated the possibility of a direct regulatory effect of gonadotrophin releasing hormone (GnRH) analogues on prostatic cancer cell growth. Here we report high affinity binding (Kd = 50 nM) of a GnRH analogue resulting in biphasic growth modulation of the human androgen-sensitive prostatic cancer cell line LNCaP. In contrast, the human androgen-insensitive prostatic cancer cell line DU145 showed low-affinity (Kd = 10 microM) binding without any biological response to the GnRH analogue. A GnRH-specific radioimmunoassay demonstrated GnRH-like immunoreactivity in the concentrated culture medium from both cell lines. Seventy-six human benign and malignant tumours were assayed following surgical resection. Nineteen of 22 (86%) malignant tumours and 49 of 54 (91%) benign tumours, exhibited high affinity GnRH-analogue binding. Fourteen of 19 (74%) malignant tumours and 17 of 49 (35%) benign tumours exhibiting high affinity binding contained GnRH-like immunoreactivity, suggesting that this system may be involved in prostatic epithelial cell growth in vivo.


Br. J. Cancer (1990), 62, 96-99 © Macmillan Press Ltd., 1990

### The effects of gonadotrophin releasing hormone analogues in prostate cancer are mediated through specific tumour receptors

A. Qayum[1], W. Gullick[2], R.C. Clayton[3], K. Sikora[1] & J. Waxman[1]

[1]*Department of Clinical Oncology and* [2]*ICRF Oncology Group, Royal Postgraduate Medical School, and* [3]*Clinical Endocrinology, MRC, Northwick Park Hospital, London, UK.*

**Summary** We have investigated the possibility of a direct regulatory effect of gonadotrophin releasing hormone (GnRH) analogues on prostatic cancer cell growth. Here we report high affinity binding ($K_d$ = 50 nM) of a GnRH analogue resulting in biphasic growth modulation of the human androgen-sensitive prostatic cancer cell line LNCaP. In contrast, the human androgen-insensitive prostatic cancer cell line DU145 showed low-affinity ($K_d$ = 10 μM) binding without any biological response to the GnRH analogue. A GnRH-specific radioimmunoassay demonstrated GnRH-like immunoreactivity in the concentrated culture medium from both cell lines. Seventy-six human benign and malignant tumours were assayed following surgical resection. Nineteen of 22 (86%) malignant tumours and 49 of 54 (91%) benign tumours, exhibited high affinity GnRH-analogue binding. Fourteen of 19 (74%) malignant tumours and 17 of 49 (35%) benign tumours exhibiting high affinity binding contained GnRH-like immunoreactivity, suggesting that this system may be involved in prostatic epithelial cell growth *in vivo*.

Cancer of the prostate is the second most common cause of malignancy among males. The treatment of prostatic cancer aims to reduce serum androgen concentrations. The long-acting agonist analogues of GnRH have been recently introduced as effective alternative therapies to orchiectomy and oestrogen treatment (Waxman, 1987). Their effect on androgen dependent cell growth is thought to be mediated through down-regulation of the pituitary-gonadal axis. GnRH agonist treatment leads to an initial increase in the serum levels of LH, FSH and androgens and this may be accompanied by tumour flare (Waxman *et al.*, 1985). However, this exacerbation of symptoms is not temporally related to changes in serum LH, FSH and androgen concentrations. The differences in the timing of these biochemical and clinical phenomena raises the possibility of a direct effect of GnRH analogues at the level of the tumour.

This present study explored the direct effects of GnRH analogues on prostatic cancer cells in culture and *in vivo*. GnRH analogue binding to human prostatic cancer cells grown in culture and biopsy samples was first examined. GnRH binding sites were characterised in cell lines and in human tumours. The biological effects of GnRH on prostatic cancer cells and a possible autocrine stimulatory role of GnRH-like peptides were investigated.

### Materials and methods

### *Cell culture*

The human androgen-sensitive prostatic cancer cell line LNCaP (isolated from lymph node metastasis; Horoszewicz *et al.*, 1983) and the human androgen-insensitive prostatic cell line DU145 (isolated from brain metastasis) were obtained from the American Type Culture Collection. Cultures were maintained in exponential growth in RPMI 1640 medium containing 10% charcoal stripped fetal calf serum and 5 $\mu g\ ml^{-1}$ insulin. The fetal calf serum used in cell culture medium was treated with dextran and charcoal to remove steroids. Charcoal 0.25% and dextran-T70 0.025% were added to the serum, which was heated at 56°C for 2 h and then centrifuged at 4,000 r.p.m. for 10 min. The pellet was discarded, serum was filtered through 0.4 μm filters and used in culture medium. The treated serum was assayed for steroid hormones by the Hammersmith Hospital Endocrine Laboratories and found to be steroid free. Cultures were incubated at 37°C in a humidified atmosphere containing 5% $CO_2$ and subcultured weekly using 0.1% EDTA to remove the cells from the plastic substratum. Under these conditions, the doubling times of DU145 and LNCaP cells were 72 and 144 h respectively.

### *GnRH binding assays*

Exponentially growing cells were harvested with 0.1% EDTA, washed twice with PBS and counted using 0.2% trypan blue as an indicator. The cells were then resuspended in assay buffer (10 mM Tris-HCl pH 7.6, 1 mM dithiothreitol, 0.3% bovine serum albumin). One million cells per tube were incubated with 200,000 c.p.m. (0.1 nM) of $^{125}$I buserelin ($^{125}$I-D-Ser (TBU)$^6$-LHRH-ethylamide) (Hoechst AG, Frankfurt), (specific activity 53,000 $kBq\ \mu g^{-1}$) with or without varying concentrations of unlabelled buserelin (0.1-100 nM). Non-specific binding was estimated in the presence of 10 μM unlabelled buserelin. After 3 h incubation at 4°C, 1 ml of ice-cold assay buffer was added and the tubes were immediately centrifuged at 2,000 *g* for 10 min. The supernatants were discarded and the pellets were washed once with ice-cold assay buffer and counted in a gamma counter. The optimal time and temperature dependence of GnRH analogue binding was determined in both cell lines.

### *Preparation of tumour cell membranes and cytosolic fractions*

After transurethral resection prostatic biopsy specimens were immediately frozen at −70°C, and subsequently cleaned and homogenised in PBS using an Ultraturrex homogenizer (Janke and Kunkel IKA Labortechnik, FR Germany) for three periods of 30 s each with an interval of 3 min on ice between each treatment. The debris and nuclear material were removed by centrifuging twice at 2,000 *g* for 10 min, discarding the pellet each time. The resultant supernatants were centrifuged at 10,000 *g* for 30 min at 4°C. The pellets were resuspended in PBS and either used immediately for binding experiments or stored frozen at −70°C. The supernatants were stored frozen at −70°C until use. The protein content was determined by Bradford's method (Bradford, 1976). Two hundred μg of membrane proteins per tube were used to assess the amount of $^{125}$I-buserelin binding.

Correspondence: J. Waxman, Department of Clinical Oncology, Hammersmith Hospital, Du Cane Road, London, W12 0HS, UK.
Received 6 November 1989; and in revised form 26 January 1990.

*Biological response studies*

Exponentially growing cells were harvested using 0.1% EDTA, counted with 0.2% trypan blue and washed once with PBS. One million cells per flask were seeded in T-25 flasks in 5 ml of RPMI 1640 medium containing 10% charcoal stripped fetal calf serum. Fresh medium containing various concentrations of buserelin with or without a 100-fold excess of the GnRH antagonist D-pGlu$^1$, D-Phe$^2$, D-Trp$^{3,6}$) GnRH (Sigma, UK) was added every third day. After 10 days the cells were harvested with 0.1% EDTA, washed once with PBS and counted with 0.2% trypan blue. Total DNA was estimated by Burton's method (Burton, 1956).

*GnRH radioimmunoassay*

The cells were cultured in RPMI 1640 medium containing 10% charcoal stripped fetal calf serum. The medium was collected after 2 weeks, without refeeding, acidified with 0.1% trifluoroacetic acid and centrifuged at 5,000 *g* for 10 min. The pellet was discarded and the supernatant was passed through C-18 Sep-Pak cartridge (Millipore, UK) (pre-wetted with methanol) followed by acidified water. The retained portion was eluted with 60% acetonitrile in water. There was 70% recovery of a control solution of buserelin added to fresh culture medium after a similar extraction procedure. The cytosol preparations from human prostatic samples were similarly concentrated. The eluates were examined for GnRH-like activity using GnRH radioimmunoassay kit (Amersham, UK). The sensitivity of the assay was 0.25 fmol per tube, inter-assay variation 7% and intra-assay variation 12%. Bradykinin, oxytocin, TRH, concentrated culture medium containing 10% dextran-charcoal heat-inactivated FCS and concentrated culture medium containing 10% normal FCS did not show any GnRH-immunoreactivity under the same conditions (Figure 1).

### Results

*GnRH receptor expression in prostatic cancer cell lines*

$^{125}$I-buserelin binding to membrane preparations from both prostatic cell lines is shown in Figure 2. The LNCaP cells showed high affinity binding of buserelin with 50% inhibition obtained at 50 nM concentration of unlabelled peptide. The binding was saturable and specific as the structurally unrelated peptides bradykinin, oxytocin and TRH did not displace labelled buserelin in binding assays. A Scatchard plot of the same data indicated a single class of binding sites with $K_d$ = 40 nM (Figure 3). The binding of buserelin to DU145 cells was inhibited only by very high concentrations of unlabelled buserelin ($K_d$ = 10 μM) indicating the presence of only very low affinity binding sites in these cells. The difference in specific binding of buserelin to both cell lines was more evident when a protease substrate (L-cystine-*bis*-(4-nitroanilide)) was included in the binding assays. This protease substrate has been shown to compete with GnRH for GnRH-degrading sites (Kuhl & Baumann, 1981; Kuhl & Taubert, 1975). The addition of this protease substrate to the assay tubes in a final concentration of 10 μM reduced the specific binding by 68% in DU145 but by only 28% in LNCaP cells (Figure 4).

**Figure 2** Binding of $^{125}$I-GnRH analogue to LNCaP (●) and DU145 (○) cells (specifically bound (B): maximum bound (BO) ratio × 100 on *y* axis) as a function of ligand concentration (Buserelin log M on *x* axis). The binding assays were carried out as described in Materials and methods. The results are expressed as the mean ± s.e.m. of triplicate measurements from a representative experiment. The $K_d$ of GnRH analogue binding in LNCaP was 50 nM, while that of DU145 cells 10 was μM.

*The biological effect of buserelin on prostatic cancer cells*

Treatment of LNCaP cells with buserelin for 10 days stimulated the growth of the cells by as much as 140% relative to the control cultures as measured both by cell numbers and total DNA content (Figure 5). This stimulatory effect was observed only at lower concentrations of buserelin (1–10 nM)

**Figure 1** GnRH-like activity detected from culture medium of LNCaP cells. The cells were cultured for two weeks and culture medium was concentrated as described in Materials and Methods. Culture medium containing 10% normal FCS (3), culture medium containing 10% dextran-charcoal treated heat-inactivated FCS (4), bradykinin (5), oxytocin (6) and TRH (7) did not show any GnRH-immunoreactivity in GnRH-RIA. The concentrated culture media from 2 (1) and 20 (2) million LNCaP cells showed the presence of 3 and 30 fmol of GnRH-like activity.

**Figure 3** Scatchard transformation of the same data shown in Figure 1 (the ratio of bound buserelin (B) to free buserelin (F) is on the *y* axis and bound buserelin in fmol is on the *x* axis) revealed that buserelin binds to a single class of binding sites in LNCaP cells of moderately high affinity ($K_d$ = 40 nM, 35,000 binding sites per cell).

**Figure 4** Specific binding of labelled buserelin (L−, D−) was decreased to 68% and (L +, D +) 28% in LNCaP (L) and DU145 (D) cells respectively when 10 μM protease substrate was added to the assay tubes. Specific binding in the absence of protease substrate was taken as 100%. Non-specific binding was determined in the presence of 10 μM unlabelled buserelin.

**Figure 5** Effect of buserelin treatment of LNCaP (circles) and DU145 (triangles) cells in culture. The cells were cultured and treated as described in Materials and methods. The cell number (open symbols) and total DNA content (filled symbols) has been expressed as percent of control and plotted as a function of buserelin concentration. Each point represents ± s.e.m. of three experiments each in triplicate.

which correlated well with the measured binding affinity. The growth of the cells was slightly inhibited at higher concentrations of GnRH analogue (100 nM). The androgen-insensitive cells did not show any response to buserelin treatment. No effects on cell growth were seen in short term culture. The stimulatory and inhibitory effects of buserelin on androgen-sensitive LNCaP cells were partially blocked when 100-fold excess of GnRH-antagonist was added to the culture medium (Figure 6).

*Prostatic cancer cells secrete GnRH-like material*

The observation that GnRH binding resulted in growth stimulation raised the possibility of the secretion of GnRH-like peptides by LNCaP cells themselves. We therefore examined this using a specific radioimmunoassay. The GnRH radioimmunoassay detected GnRH-like immunoreactivity in concentrated medium collected after two weeks culture of both cell lines. The concentrated culture media from 2 and 20 million LNCaP cells showed the presence of 3 and 30 fmol of GnRH-like immunoreactivity respectively (Figure 1). Twenty million DU145 cells cultured for 2 weeks secreted 35 fmol GnRH-like immunoreactivity. The elution profile of this immunoreactive peptide on high performance liquid chromatography was identical to native GnRH.

**Figure 6** The effect of the GnRH antagonist on buserelin-induced growth modulation in LNCaP cells. The cells were cultured and treated with indicated concentrations of buserelin with or without a 100-fold excess of GnRH antagonist. The cell numbers have been expressed as per cent of control and plotted as function of buserelin concentration. Each point represents ± s.e.m. of triplicate from a representative experiment. ■, buserelin only; ●, antagonist only; ▲ buserelin + antagonist.

*GnRH receptors and GnRH immunoreactive peptides are present in prostatic biopsy specimens*

GnRH analogue binding was assessed on membrane preparations from 76 benign and malignant prostate tumours. Forty-nine of 54 (91%) benign tumours exhibited high affinity binding ($K_d$ = 46 ± 43 nM, capacity 390 ± 218 fmol $mg^{-1}$ protein) and 19 of 22 (86%) malignant tumours showed high affinity binding ($K_d$ = 10 ± 14 nM, capacity 301 ± 180 fmol $mg^{-1}$ protein). The GnRH radioimmunoassay showed GnRH-like peptides to be present in 17 of 49 (35%) benign tumours exhibiting high affinity ligand binding sites, but in none without binding. Fourteen of 19 (74%) malignant tumours having high affinity ligand binding sites and two of three showing no ligand binding contained GnRH-lilke peptides (Table I). The elution profile on high performance liquid chromatography of this peptide was identical to native GnRH.

### Discussion

Extrapituitary receptors for GnRH have been described in normal testis and ovary (Clayton *et al.*, 1979, 1980). There have also been reports of the presence of GnRH-binding sites in breast cancer cells (Eidne *et al.*, 1987), pancreatic tumours (Szende *et al.*, 1989) and induced rat prostatic cancers (Kadar *et al.*, 1988). The binding of GnRH to breast cancer cells has been shown to result in growth modulation (Millar *et al.*, 1985; Eidne *et al.*, 1987). In this present study the observation of high affinity binding sites in prostatic cancer cell lines and prostatic biopsy specimens suggests that GnRH and its analogues may exert their effects directly on tumour tissue. However, the biological effect observed in short term culture at therapeutic concentrations (1−10 nM) of GnRH analogue is stimulation of growth rather than inhibition. The initial direct stimulatory effect of GnRH analogues on prostatic cancer cells may therefore explain the lack of a temporal correlation of hormonal change with the clinical observation of tumour flare. It is possible that the long-term and continued occupancy of its binding sites by GnRH-analogues leads to desensitisation and down-regulation of receptors resulting in growth inhibition.

**Table I** Fresh frozen, transurethrally resected benign and malignant prostatic tissues examined for GnRH binding

| | No. | $K_d \times 10^{-9}$ M | Capacity (fmol mg$^{-1}$) | GnRH-RIA + ve | GnRH-RIA − ve | GnRH activity fmol g$^{-1}$ tissue |
|---|---|---|---|---|---|---|
| *Benign prostatic hypertrophy* | | | | | | |
| GnRH-R + ve | 49/54 (90%) | 46±43 – | 390±218 – | 32/49 (65.5%) | 17/49 (34.7%) | 26.16±20.2 |
| GnRH-R − ve | 5/54 (9.2%) | | | 5/5 (100%) | 0/5 | 33.12±24.1 |
| *Prostatic cancer* | | | | | | |
| GnRH-R + ve | 19/22 (85%) | 10±14 | 301±180 | 5/19 (26.3%) | 14/19 (73/7%) | 77±71.4 |
| GnRH-R − ve | 3/22 (13.6%) | – | – | 1/3 (33.3%) | 2/3 (66.6%) | 100 |

The cytosolic preparations from tissues were concentrated and assayed for GnRH-like activity in GnRH-RIA. Results are summarised from 76 prostatic tissues.

The finding of specific GnRH-binding sites in prostatic cancer cells which modulate growth, and the observation of the secretion of GnRH-like peptides by these cells, suggests that GnRH-like peptides may play an autocrine stimulatory role in this system. It is not yet known whether GnRH itself has mitogenic activity nor whether it has synergy with other stimulatory growth factors, and this requires further investigations. The results of our study provide impetus for the development of GnRH antagonists for use in prostatic cancer treatment.

We thank Dr J. Sandow (Hoechst AG, Frankfurt) for the gift of labelled and unlabelled buserelin, and Dawn Butler for secretarial assistance.

### References

BRADFORD, M.M. (1976). A rapid and sensitive method for the quantitation of microgram quantities of protein utilizing the principle of protein-dye binding. *Anal. Biochem.*, **72**, 248.

BURTON, K. (1956). A study of the conditions and mechanisms of the diphenylamine reaction for the colorimetric estimation of deoxyribonucleic acid. *Biochem. J.*, **62**, 315.

CLAYTON, R.N., HARWOOD, J.P. & CATT, K.J. (1979). Gonadotrophin-releasing hormone analogue binds to luteal cells and inhibits progesterone production. *Nature*, **282**, 90.

CLAYTON, R.N., KATIKINENI, M., CHAN, V., DUFAU, M. & CATT, K. (1980). Direct inhibition of testicular function by a gonadotrophin-releasing hormone: mediation by specific gonadotrophin-releasing hormone receptors in interstitial cells. *Proc. Natl Acad. Sci. USA*, **77**, 4459.

EIDNE, K.A., FLANAGAN, C.A., HARRIS, N.S. & MILLAR, R.P. (1987). GnRH binding sites in human breast cancer cell lines and inhibitory effect of GnRH antagonists. *J. Clin. Endocrinol. Metab.*, **64**, 425.

HOROSZEWICH, J.S., LEONG, S.S., KAWINSKI, E. *et al.* (1983). LNCaP model of human prostatic carcinoma. *Cancer Res.*, **43**, 1809.

KADAR, T., REDDING, T.W., BEN-DAVID, M. & SCHALLY, A.V. (1988). Receptors for prolactin, somatostatin and LHRH in experimental prostatic cancer after treatment with analogues of LHRH and somatostatin. *Proc. Natl Acad. Sci. USA*, **85**, 890.

KUHL, H. & BAUMANN, R. (1981). New aspects of the physiological significance of LHRH receptors of pituitary plasma membranes. *Acta Endocrinol.*, **96**, 36.

KUHL, H. & TAUBERT, H.D. (1975). Inactivation of luteinizing hormone-releasing hormone by rat hypothalamic L-cystine arylamidase. *Acta Endocrinol.*, **78**, 634.

MILLAR, W.R., SCOTT, W.N., MORRIS, R., FRASER, H.M. & SHARPE, R.M. (1985). Growth of human breast cancer cells inhibited by a luteinizing hormone-releasing hormone agonist. *Nature*, **313**, 231.

SZENDE, B., ZALATNAI, A. & SCHALLY, A.V. (1989). Programmed cell death (apoplosis) in pancreatic cancers of hamster after treatment with analogs of both luteinizing hormone-releasing hormone and somatostatin. *Proc. Natl Acad. Sci. USA*, **86**, 1643.

WAXMAN, J. (1987). Gonadotrophin hormone releasing analogues open new doors in cancer treatment. *Br. Med. J.*, **295**, 1084.

WAXMAN, J.H., MAN, A., HENDRY, W.F. *et al.* (1985). Importance of early tumour exacerbation in patients treated with long acting analogues of gonadotrophin releasing hormone for advanced prostatic cancer. *Br. Med. J.*, **291**, 1387.